# The volume of general surgery emergency cases in a government hospital during the COVID-19 pandemic and two other periods: a comparative, retrospective study

**DOI:** 10.1186/s12893-021-01131-4

**Published:** 2021-03-08

**Authors:** Ibrahim Abu Shakra, Maxim Bez, Samer Ganam, Rola Francis, Amir Muati, Amitai Bickel, Fahed Merei, Ziv Talmi, Khatib Kamal, Eli Kakiashvili

**Affiliations:** 1 Department of Surgery A, Galilee Medical Center, 22100 Nahariya, Israel; 2 Israel Defense Forces, Medical Corps, Ramat Gan, Israel; 3 Faculty of Medicine in the Galilee, Bar-Ilan University, Safed, Israel

**Keywords:** COVID-19, SARS-CoV-2, Emergency surgery, General surgery

## Abstract

**Background:**

During March and April 2020, reductions in non-COVID-19 hospital admissions were observed around the world. Elective surgeries, visits with general practitioners, and diagnoses of medical emergencies were consequently delayed.

**Objective:**

To compare the characteristics of patients admitted to a northern Israeli hospital with common surgical complaints during three periods: the lockdown due to the COVID-19 outbreak, the Second Lebanon War in 2006, and a regular period.

**Methods:**

Demographic, medical, laboratory, imaging, intraoperative, and pathological data were collected from electronic medical files of patients who received emergency treatment at the surgery department of a single hospital in northern Israel. We retrospectively compared the characteristics of patients who were admitted with various conditions during three periods.

**Results:**

Patients’ mean age and most of the clinical parameters assessed were similar between the periods. However, pain was reportedly higher during the COVID-19 than the control period (8.7 vs. 6.4 on a 10-point visual analog scale, P < 0.0001). During the COVID-19 outbreak, the Second Lebanon War, and the regular period, the mean numbers of patients admitted daily were 1.4, 4.4, and 3.0, respectively. The respective mean times from the onset of symptoms until admission were 3, 1, and 1.5 days, P < 0.001. The respective proportions of surgical interventions for appendiceal disease were 95%, 96%, and 69%; P = 0.03.

**Conclusions:**

Compared to a routine period, patients during the COVID-19 outbreak waited longer before turning to hospitalization, and reported more pain at arrival. Patients during both emergency periods were more often treated surgically than non-operatively.

## **Background**

During the months March and April 2020, countries around the world enforced social containment rules, including public commute restrictions and the closure of “non-essential” public places, as means to reduce the spread of the COVID-19 pandemic [1]. In the same time many hospitals had reported a decline in the volume of emergency department (ED) visits, including for serious surgical conditions among other life-threatening diagnoses; possibly reflecting reluctance to seek medical care due to the high risk of infection from the novel virus. Despite the decrease in ED visits most studies reported no change in the rate of admissions. This discrepancy was suggested to be caused by the higher acuity of patients presented to the ED, in some cases resulting from a delayed presentation [2–4]. Although the changes in ED utilization are similar across multiple studies, there are contradicting reports regarding the rate of emergency surgeries during the pandemic [3, 5].

The first case of COVID-19 in Israel was discovered in Israel on February 22nd, followed by an increase in case number during the month of March. To combat the pandemic, the Israeli government had enforced restrictions similar to other countries, and on March 19th had declared a state of emergency. During the state of emergency all Israeli residents were to remain within 100-meter radius of their home except for essential needs and medical emergencies. Another state of emergency, though different in nature, was declared in Israel during the Second Lebanon War in 2006. On July 15th the Israeli Minister of Defense declared the state of emergency, which lasted one month.

In this study we aimed to describe the effect of different states of emergency on time to treatment and method of treatment in surgical patients admitted to the surgical department. The COVID pandemic period and the 2006 Lebanon War period were compared to a control period, hypothesizing that the state of emergency periods will lead to a later ED presentation and will therefore result with a higher rate of emergent surgical treatment.

## Methods

This single center retrospective study included patients who were admitted to Galilee Medical Center, Israel, with surgical emergencies, during three periods. The first period is during the COVID-19 pandemic outbreak, when a countrywide lockdown was enforced from March 1, 2020 to April 19, 2020 (50 days). The second period refers to the Second Lebanon war, between July 12, 2006 and August 14, 2006 (34 days). Both these periods were defined as states of emergency by the Israeli government. For the establishment of a control group, the third period was defined as August 1, 2019 to December 31, 2019 (153 days). The study was approved by the institutional Helsinki Committee.

### Data collection

Data were collected from electronic medical files of all the patients who received emergency treatment at the surgery department. The files were reviewed for demographic data (gender, age) medical data (chronic comorbidities, associated illnesses, the elapsed time since the start of symptoms, the surgical approach, the type and duration of treatment, hospital stay, perioperative complications and pain assessed by standard 10-point visual analogue scale [VAS]), and laboratory, pathological, imaging analysis, and intraoperative findings. Pain was self-reported by the patient using a graphical ruler [6]. To investigate possible differences in clinical presentation and treatment between the state of emergency periods and the control period, characteristics of patients admitted to the surgical department were examined with the following conditions: intestinal diseases (intestinal obstruction, volvulus, intestinal perforation, diverticulitis, and mesenteric ischemia), diseases of the appendix (appendicitis and appendicular abscess), biliary diseases (acute cholecystitis, cholangitis, and pancreatitis), gastric and duodenal ulcers and perforations, abscesses (perineal, pilonidal, and gluteal), gastrointestinal bleeding, and incarcerated or strangulated hernias (including inguinal, femoral, ventral, and umbilical hernias).

### Statistical analysis

Means, standard deviations, and medians were calculated for all the variables examined. Continuous variables were tested for normal distribution using the Kolmogory-Smirnov test, and groups were compared using ANOVA or the Kruskal-Wallis test. The Chi squared test was used to compare categorical variables between groups. All statistical analyses were done using SPSS ver. 25.

## Results

This study included a total of 677 patients admitted to the surgical department; 71 during the COVID-19 outbreak, 149 during the 2006 s Lebanon War, and 457 during the control period. The mean numbers of patients per day for the respective periods were 1.4, 4.4, and 3.0. Most patients were admitted due to biliary disease, abscess, intestinal disease, or acute appendicitis: 28.4, 24.1%, 21.0%, and 15.2% respectively (Table 1).

**Table 1:** Reasons for presentation at the surgical department during three periods of time

	Control (n = 457)	COVID-19 (n = 71)	Wartime (n = 149)	Total (n = 677)
Gastrointestinal bleeding	19 (4.1%)	1 (1.4%)	6 (4%)	26 (3.8%)
Hernia	14 (3.0%)	4 (5.6%)	18 (12.0%)	36 (5.3%)
Intestinal diseases	105 (22.9%)	11 (15.4%)	26 (17.4%)	142 (21.0%)
Other diseases	2 (0.1%)	0	6 (4.0%)	8 (1.2%)
Abscess	116 (25.3%)	21 (29.5%)	26 (17.4%)	163 (24.1%)
Acute appendicitis	55 (12.0%)	19 (26.8%)	29 (19.5%)	103 (15.2%)
Biliary diseases	140 (30.0%)	13 (18.0%)	39 (26.0%)	192 (28.4%)
Perforation and ulcer	3 (0.6%)	2 (2.8%)	2(1.3%)	7 (1.0%)

The mean age of the patients did not differ between the periods of COVID-19, the Second Lebanon war, and the control groups (48.9 ± 22.2, 51.9 ± 20.7, and 52.1 ± 20.9, % respectively, P = 0.48; Table 2). During the COVID-19 period, the percentage of patients presenting at the surgical department with diabetes was lower and the percentage with COPD was higher than during the other periods examined. Clinical and laboratory variables did not differ between the groups including heart rate, mean arterial pressure, body temperature, white blood cell count (WBC) and C-reactive protein (CRP). However, pain was rated higher during the COVID-19 than during the control period (8.7 vs. 6.4, P < 0.0001); VAS data were not available for the 2006 Lebanon war group. The time from the onset of symptoms until admission at the surgical ward was longest during the COVID-19 period (3 days), and shortest during wartime (1 day), P < 0.005. In both state-of-emergency periods, 63% of the patients who were admitted received surgical treatment compared to only 36% in the control group (P < 0.001).

**Table 2:** Demographic and clinical characteristics of patients who presented at the surgical department during three periods of time

	Control (n = 457)	COVID-19 (n = 71)	Wartime (n = 149)	P value
Age, years	52.1 ± 20.9	48.9 ± 22.2	51.9 ± 20.7	P = 0.48
Gender				
Male	257 (56)	42 (59%)	92 (62%)	P = 0.48
Female	200 (44%)	29 (41%)	57 (38%)	
Heart failure	58 (13%)	9 (13%)	29 (20%)	P = 0.11
Diabetes	118 (26%)	5 (7%)	23 (15%)	**P** ^**1,2**^**<0.01**
Kidney failure	20 (4%)	7 (10%)	8 (6%)	P = 0.15
COPD	43 (9.2%)	15 (21.1%)	23 (15.8%)	**P** ^**1,3**^ **<0.05**
Asthma	43 (9.2%)	8 (11.3%)	24 (16.4%)	**P** ^**2**^ **= 0.02**
Hemoglobin	13.2 ± 2.2	13.6 ± 2.3	13.1 ± 2.1	P = 0.16
White blood cell count	11.4 ± 3.7	11.5 ± 4.0	11.85 ± 4.30	P = 0.41
CRP, median, IQR	16.3 [4.9–57.8]	13.4 [5.1–54.0]	12.8 n = 2	P = 0.58
Body temperature	36.8 ± 0.37	36.7 ± 0.22	36.8 ± 0.59	p = 0.98
pH	7.37 ± 0.06	7.35 ± 0.05	7.51 n = 1	P = 0.60
Heart rate	80.0 ± 12.1	80.1 ± 8.9	81.7 ± 14.5	P = 0.65
Pain, according to VAS	6.39 ± 1.17	8.72 ± 0.81	N/A	**P < 0.001**
Creatinine	0.99 ± 0.64	0.89 ± 0.24	1.06 ± 0.42	P = 0.15
BMI	27.9 ± 5.3	27.7 ± 4.5	23.2 n = 1	P = 0.60
Time from symptoms, median, IQR	1.5 [1–3]	3[ 1–4]	1 [ 0.42–1.5]	**P** ^**1,2,3**^**<0.001**
Treatment				**P** ^**1,2**^ **<0.001**
Surgery	166 % (36%)	45 (63%)	93 (63%)	P = 1.00
Invasive	9 (2%)	0	3 (2%)	P = 1.00
Invasive and conservative	10 (2%)	0	4 (3%)	*P ^**1** ^ **= 0.0004**
Conservative	*272 (60%)	*26 (37%)	48 (32%)	**P** ^**2** ^ **< 0.0001**
Dementia (cognitive status)	23 (5%)	8 (11.3%)	10 (6.7%)	P = 0.054

P1 = Control vs. COVID-19 period, P2 = Control vs. Lebanon War period, P3 = COVID-10 vs. Lebanon War period

*COPD* chronic obstructive pulmonary disease, *CRP* C-reactive protein, *VAS* visual analogue scale, *BMI* body mass index, *IQR* interquartile range

Examining the surgical conditions separately, the differences observed between the groups in time from onset of symptoms until admission remained constant. For example, among patients with biliary disease, the median time until admission was 2 days in the COVID-19 group, 1 day in the 2006 Lebanon war group and 1.5 days in the control group (P = 0.021). The same pattern was observed among patients with appendiceal disease, with medians of 2 days, 0.5 days, and 1 day, respectively (P < 0.001).

The method of treatment of certain conditions differed between the time periods. For patients with appendiceal disease, more surgical interventions were performed during the state-of-emergency periods than during the control period (95, 96%, and 69% during COVID-19, the Second Lebanon war and the control period, respectively; P = 0.03). During the Second Lebanon war, 46% of the patients with biliary disease underwent surgical treatment compared to 4% during the control period and 8% during the COVID-19 period.

Eleven patients presented with intestinal diseases, during the COVID-19 pandemic compared to 26 during the Lebanon war and 105 patients during the control period; the respective average admissions per day were 0.22, 0.76., and 0.69. During the COVID-19 pandemic, patients with intestinal diseases were symptomatic for a longer period and arrived to the hospital within a median of 3 days compared to 0.75 days during the war and one day during the control period; P < 0.05. All the patients with gastrointestinal diseases were treated conservatively, with the exception of one patient who underwent surgery during the COVID-19 pandemic; no differences were observed between the periods.

## Discussion

This retrospective study aimed to examine the characteristics of patients treated in the surgical ward of The Galilee Medical Center during the COVID-19 outbreak. The focus was the time from onset of symptoms until treatment, and the treatment method. We compared patients treated during the COVID-19 outbreak to those treated during the 2006 Lebanon war and to a control group. To the best of our knowledge, this is the first study that compares such periods.

The main finding of this study is that patients during the COVID-19 outbreak waited longer before turning to hospitalization than did patients during a routine period; and the waiting in both periods was longer than during the Second Lebanon war. This finding highlights differences in patient behavior during two periods of national state of emergency. In addition, to presenting later, the COVID-19 group was in more pain than the control group, according to VAS scores. Moreover, in both the COVID-19 outbreak and the Second Lebanon war, patients were more often treated surgically than non-operatively, compared to the control group. This is a novel finding, since while other studies have shown a decrease in the number of patients undergoing surgical treatment during the COVID pandemic [3], here we show higher rate of patients receiving surgical treatment. This corresponds with similar findings of higher rate of complicated appendicitis during the pandemic.

During the COVID-19 period, the percentage of patients presenting at the surgical department with diabetes was lower and the percentage with COPD was higher than during the other periods examined. This finding might be the result of younger and healthier patients trying to avoid the hospital during the COVID-19 outbreak, as is evident from the decline in the total number of emergency department (ED) visits during the outbreak.

The longer time from onset of symptoms to admission in the COVID-19 group can be explained by patients’ concerns of contracting the virus in the ED. Accordingly, the number of admissions was lower than during the other periods. This is consistent with reports from other countries, where the number of ED visits greatly declined during lockdown.[7, 8] On the other hand, the shorter time to admission in the Second Lebanon war group could be explained by a different case mix, with a higher emergency severity index. This is similar to the findings of Makhlouf-Obermeyer et al. who examined ED admissions during weeks in which a violent event occurred [9]. Nevertheless, we cannot confirm this hypothesis due to the lack of data regarding the emergency severity index.

Despite the greater time to admission during the state of emergency periods, the treatment method was more aggressive than during the control group. However, other characteristics did not differ significantly between the state of emergency and the control groups, suggesting that the emergency situation affected the surgical decision making. The higher rates of comorbidities during the state of emergency periods also support this conclusion, due to the greater possibility of intraoperative and postoperative complications and the tendency to prefer non-operative treatment in such patients.

A possible explanation for the higher rates of surgical treatment during the state of emergency periods is the greater severity of the medical conditions, a parameter that we were not able to assess directly. As suggested above, it is possible that only patients who were more ill presented to the ED during the COVID-19 lockdown. Though clinical parameters between patients in the lockdown period and the control period were similar, patients who presented during the COVID-19 outbreak reported higher levels of pain. The association between the intensity of pain and the severity of the underlying condition is well known [10] and may lead a surgeon to prefer surgical treatment over non-operative treatment. As the objective parameters were similar between the periods, including age and vital signs, the state of emergency itself may have exacerbated the pain perceived by the patients. Prior research has linked anxiety state and acute pain, and recently published papers have linked the COVID-19 outbreak to a higher level anxiety [11–14]. Pain evaluation is a routine parameter filled for each patient in our department, though its documentation started only in 2010. Therefore, we do not have data regarding the war period. It is likely that patients during the Second Lebanon war also presented to the ED with greater pain, though data are not available.

Another possible explanation for the higher rate of surgery during the COVID-19 period is that the state of emergency itself affected decisions to perform surgery. The benefit of surgical treatment and the risk of non-operative treatment have been identified as the highest predictors of surgery.[15, 16] Furthermore, Szatmary et al. found that surgeons with less surgical experience were more likely to assess higher non-operative risk and thus opted to perform surgery more often.[16] We speculate % that the uncertainty regarding the possibility to perform surgery during the hospitalization, due to resource prioritization may have contributed to the higher rates of surgery.

This study has a number of limitations due to its retrospective design. Data were not available equally for all the periods, such as the absence of pain reports during the Second Lebanon War. Moreover, hospital admissions may have been affected by differences due to the seasons of the periods assessed. Treatments may have been affected by changes in clinical practice and decision making during the 14 years that lapsed between the earliest and the latest periods. Thus the data regarding the 2006 Lebanon War should be taken in perspective to the 14-year difference between the periods, during which changes occurred in hospital and international protocols. In addition, this study covers a relatively short period of time, which in result led to the inclusion of a small number of patients. Due to these limitations, there is not enough evidence to reach clear conclusions regarding the patients.

## Conclusions

In conclusion, this study shows that patients seek medical attention at a different stage of their illness in states of national emergencies and are more likely to be treated surgically. This observation may be due to a number of factors, mostly such that result in higher rate of more complicated cases presenting to the ED, though the state of emergency itself could also influence the decision making. During the current COVID-19 outbreak the patients refrained from seeking early medical attention, possibly due to the perception that hospitals are a place with high risk for contracting the novel virus. This behavioral change poses risk for patients with surgical emergencies, and may lead to late morbidity and mortality. Further research is needed, though health care systems should consider addressing this issue in order to minimize the effect of the COVID pandemic on public health.

## Data Availability

The authors are responsible for the data described in the manuscript and assure full availability of the study material. Data is available on request from the corresponding author.

## Abbreviation

COVID-19: Coronavirus disease 2019
